# Tina Musuya: preventing violence against women

**DOI:** 10.2471/BLT.22.030322

**Published:** 2022-03-01

## Abstract

Tina Musuya talks to Tatum Anderson about her work in community-led reforms to prevent violence against women.

*Q: How did your feminist world view develop?*

A: I was born and raised in Kampala, Uganda. My dad was a high-ranking, authoritarian police officer but very progressive, very accepting of us, his four daughters. My mum was more traditional. When I refused to do the dishes or other chores, she used to say, “What kind of a wife are you going to be?” I never saw myself fitting into the housewife role and I decided to study very hard. Luckily, I was educated in girls’ schools, where our potential was nurtured and we were encouraged to realize our ambitions. It was only when I went to university, that I started to experience gender discrimination. It made me and my female friend work even harder, to always be prepared, to go to lectures and seminars and participate in discussions as a way of countering the gender stereotypes.

*Q: When did you start focusing on the specific issue of violence against women?*

A: It became personal for me because of my elder sister. This was in the early 2000s and she was experiencing a lot of violence from her husband. She would leave him and go back. I couldn’t understand that. One time he beat her so badly that I had to leave a job interview to go and pick her up. She told me that her son had asked why they couldn’t just leave, because he was afraid his father was going to kill her. That was when I saw the job being advertised at CEDOVIP in Kampala. I applied and started working there in 2003. That's where I got my education on violence against women, the issues of power that drive it, and how to prevent it.

*Q: How prevalent is violence against women in Uganda?*

A: It has been estimated that almost six women in every 10 experience intimate partner violence, reflecting societal norms that encourage men to devalue and control women. There is also a culture of silence here, a tendency to turn a blind eye to domestic abuse, again underpinned by an acceptance of the idea that women must be controlled and disciplined by men. With specific regard to sex, women are treated as sex objects and men are encouraged to have sex with whomever they choose, so sexual abuse and harassment in public spaces is a big problem. At the same time women are expected to be chaste and are generally judged to be responsible for the way men behave. So the victims of violence and sexual harassment and rape are often blamed for what happened to them. Women are also at risk of sexual assault in the home because marital rape is not even recognized under the law. Because she got married, the wife is assumed to have given her consent to sex whenever and wherever her husband chooses. Other aspects of the problem include child marriage, human trafficking, and female genital mutilation – all of which is embedded in gender norms and unequal power relations.

“The victims of violence and sexual harassment and rape are often blamed.”

*Q: How did you go about tackling these problems in CEDOVIP?*

A: By getting out into the Kawempe Division of Kampala. In the beginning I worked with a team of over 130 men and women activists who, on a daily basis, engaged groups of community members raising awareness, and supporting community-led activism to prevent domestic violence. The work was based on a resource guide developed by Raising Voices, a Ugandan NGO, and it was that work that also informed the development of SASA! which we piloted in 2007.

*Q: Can you tell us about SASA!, starting with why there is an exclamation point at the end of the name?*

A: (laughing) It is there to make you wake up! And to galvanize you into action! The word “sasa” means “now” in Kiswahili and it is appropriate because now is the time to prevent violence against women. It is also an acronym for the four phases of the SASA! approach – Start, Awareness, Support, Action.

*Q: Can you say more about that?*

A: Basically, SASA! is about changing people’s perceptions of women by using community activists. In the Start phase we select men and women who are committed to changing things, and train them in the key concepts and get them to reflect on the way power imbalances affect their own lives and the community. Then, in the Awareness phase, we send them out into the community to raise awareness and to start discussion. The basic idea is that people who come from the community can influence the community. If you know me, and I know you, we can discuss these ideas and you are more likely to be influenced by me than you would be by someone coming from the government or from the police, for example. Activists reach out to people within their own networks, friends, neighbours, colleagues within the communities. And it’s not all talk. They also interact through games and public events such as participatory community theatre. Importantly, the process has to be intensive and to a certain degree challenging without being confrontational. The intention in the awareness phase is to make people uncomfortable about the status quo and to build their activism spirit to do something about it in their own relationships and within their community.

Once people are aware, we start the Support phase to help people develop necessary skills to act on their awareness. This involves different activities. For example helping couples to make the necessary changes in how they relate to each other or helping someone to negotiate for better treatment from their partner or to challenge a neighbour they see beating their child. We also help people hold perpetrators to account and work on transforming the institutions that respond to the cases so that they are more survivor-centred.

*Q: How do you draw institutions into the conversation?*

A: We engage with opinion leaders in the police and local government or faith-based groups and in the health system. For example, we have done a lot of work on influencing practice within law enforcement institutions, encouraging police to be sensitive to the needs of the victims and survivors of interpersonal and sexual violence and female genital mutilation.

We run training sessions about how to listen in cases of domestic violence, how to investigate and how to refer victims for further support. I developed protocols such as police interview guides for cases of domestic violence and also issued policy guidelines to police officers on domestic violence.

We have also worked on addressing challenges faced in mediation to focus on survivor safety and perpetrator accountability. Unfortunately, mediators here often blame the wife for the violence. A husband may say she burned the food or gossips with neighbours and the mediator will tell her not to burn the food or gossip to avoid future beatings. Often the woman has to sit on the ground in the meetings while the husband sits on a chair.

“People […] from the community can influence the community.”

*Q: In SASA! you use the term responsibility meetings. What does that mean?*

A: The default position in mediation tends to be to find some way to reconcile, but you sometimes reach a point where the mediation is not working. In responsibility meetings we shift the position to prioritize the woman’s safety and this may include risk assessment, and where necessary helping the woman. We do a lot of work making sure referral networks are in place, training people to identify those who need help and make appropriate referrals to those who can help. We also introduce stakeholders in the referral networks to the larger community at public events and have a referral directory so people know where to seek help if someone is in need.

*Q: Does the community get a chance to challenge those who fail to act on instances of violence?*

A: Absolutely. We have accountability forums where we bring members of the formal justice system to the public events to interface with the community. They can explain the process of justice, and what is required. And the community can give them feedback about how they're experiencing that justice system.

*Q: You referred to the Action phase. What is that?*

A: When the community takes over and we step back. In many ways it is the most satisfying phase. When people begin to see changes happening and feel empowered. In the Action phase they begin to set up their own rules about what is acceptable behaviour within their own households and the broader community. For example, in Kampala some groups of boda-boda (motorcycle taxi) drivers have drawn up codes of conduct. If a driver sexually harasses clients, or even women passing by the taxi park, he can be suspended and made to pay a fine or even be reported to the authorities.

*Q: How do you know SASA! works?*

A: In terms of documented evidence, a randomized controlled trial was conducted between 2007 and 2012 in eight communities in Kampala which demonstrated benefits including a 50% drop in the trial group’s previous year’s experience of physical interpersonal violence compared with the control group. But we can also see the change in the communities where we have a presence. For example, we see landlords who put violence prevention non-renewal provisions in their tenancy agreements. We see village leaders and neighbours actively engaging with new members to their neighbourhoods. We see communities challenging the police and calling for updates on cases. Since 2014, SASA! has been rolled out in 15 African countries, as well as in countries in the Caribbean, the Middle East, South-East Asia and the South Pacific. That doesn’t mean that everyone is happy about it, of course.

*Q: What do you mean?*

A: Some groups want to maintain the patriarchal control over women. They complain that men and boys have become vulnerable because of women’s empowerment. There are also religious fundamentalists who preach female subordination or say human rights and feminist initiatives are breaking up families. They often tell women to stay submissive and dress properly, so they do not get raped! So there is a lot more work to be done and it needs to be done now.

